# Triheptanoin in patients with long-chain fatty acid oxidation disorders: clinical experience in Italy

**DOI:** 10.1186/s13052-024-01782-y

**Published:** 2024-10-07

**Authors:** Francesco Porta, Arianna Maiorana, Vincenza Gragnaniello, Elena Procopio, Serena Gasperini, Roberta Taurisano, Marco Spada, Carlo Dionisi-Vici, Alberto Burlina

**Affiliations:** 1 Department of Paediatrics, University of Turin, Turin, Italy; 2 Division of Metabolic Diseases and Hepatology, Bambino Gesù Children‛s Hospital IRCCS, Piazza S. Onofrio 4, 00165 Rome, Italy; 3 Division of Inherited Metabolic Diseases, Department of Women and Children‛s Health, Reference Centre for Expanded Newborn Screening, University Hospital, Padua, Italy; 4 Metabolic and Neuromuscular Unit, Meyer Children‛s Hospital IRCCS, Florence, Italy; 5 Department of Paediatrics, Fondazione IRCCS San Gerardo Dei Tintori, Monza, Italy

**Keywords:** Inherited metabolic disorders, Long-chain fatty acid oxidation disorders, Medium-chain triglyceride oil, Triheptanoin

## Abstract

**Background:**

Long-chain fatty acid oxidation disorders (LC-FAOD) are rare and potentially life-threatening diseases that cause deficient energy production and accumulation of toxic metabolites. Despite dietary management, adherence to maximum fasting guidelines, restricted long-chain triglyceride intake and supplementation with medium-chain triglyceride (MCT) oil (current standard of care), most patients experience recurrent decompensation episodes that can require hospitalisation. Herein, we analysed the effectiveness and safety of triheptanoin (a highly purified, synthetic medium odd-chain triglyceride) treatment in a cohort of Italian patients with LC-FAOD.

**Methods:**

This retrospective, nationwide study included nine patients with LC-FAOD who switched from standard therapy with MCT oil to triheptanoin oral liquid. Data were collected between 2018 and 2022. Clinical outcome measures were the number and duration of intercurrent catabolic episodes and number and duration of metabolic decompensation episodes requiring hospitalisation. Creatine kinase (CK) levels and treatment-related adverse effects were also reported.

**Results:**

Patients were provided a mean ± standard deviation (SD) triheptanoin dose of 1.5 ± 0.9 g/kg/day in four divided administrations, which accounted for 23.9 ± 8.9% of patients’ total daily caloric intake. Triheptanoin treatment was started between 2.7 and 16 years of age and was continued for 2.2 ± 0.9 years. The number of intercurrent catabolic episodes during triheptanoin treatment was significantly lower than during MCT therapy (4.3 ± 5.3 vs 22.0 ± 22.2; *p* = 0.034), as were the number of metabolic decompensations requiring hospitalisation (mean ± SD: 2.0 ± 2.5 vs 18.3 ± 17.7; *p* = 0.014), and annualised hospitalisation rates and duration. Mean CK levels (outside metabolic decompensation episodes) were lower with triheptanoin treatment versus MCT oil for seven patients. No intensive care unit admissions were required during triheptanoin treatment. Epigastric pain and diarrhoea were recorded as adverse effects during both MCT and triheptanoin treatment.

**Conclusions:**

The significant improvement in clinical outcome measures after the administration of triheptanoin highlights that this treatment approach can be more effective than MCT supplementation in patients with LC-FAOD. Triheptanoin was well tolerated and decreased the number of intercurrent catabolic episodes, metabolic decompensation episodes requiring hospitalisation, and the annualised rate and duration of hospitalisations.

## Background

Long-chain fatty acid oxidation disorders (LC-FAOD) are rare, autosomal recessive, potentially life-threatening disorders leading to deficient energy production and accumulation of toxic metabolites [1, 2]. Deficiencies in any of the long-chain fatty acid β-oxidation enzymes can cause metabolic disorders, such as very long-chain acyl-coenzyme A dehydrogenase deficiency (VLCADD), long-chain hydroxyacyl-coenzyme-A dehydrogenase deficiency (LCHADD) and mitochondrial trifunctional protein deficiency (MTPD), or as disorders of carnitine transport, such as carnitine palmitoyltransferase type 1 deficiency (CPT1D), carnitine palmitoyltransferase type 2 deficiency (CPT2D) and carnitine-acylcarnitine translocase deficiency (CACTD) [2].

Common signs and symptoms in LC-FAOD in the first weeks of life include cardiac arrhythmias, hypoglycaemia and sudden death; later in infancy and early childhood, the disease presents as liver- or cardiac-related symptoms or skeletal muscle dysfunction, including fasting or stress-related hypoketotic hypoglycaemia, hyperammonaemia (or Reye-like syndrome), conduction abnormalities, arrhythmias, dilated or hypertrophic cardiomyopathy and muscle weakness or fasting- and exercise-induced rhabdomyolysis [3]. Patients with adolescent or adult-onset LC-FAOD predominantly experience muscular symptoms, including rhabdomyolysis and cardiomyopathy. Episodes of metabolic decompensation are usually triggered by fasting, intercurrent febrile illnesses or prolonged exercise [4]. Different from other LC-FAOD, most patients with LCHADD and MTPD also present with peripheral sensory-motor polyneuropathy and pigmentary retinopathy during the course of the disease [3], and more rarely with hypoparathyroidism [1].

The current standard of care for LC-FAOD is dietary management, including maximum fasting guidelines (under healthy steady-state conditions and according to age), restricted long-chain triglyceride (LCT) intake and supplementation with medium-chain triglyceride (MCT) oil [5]. Despite this management approach, most patients will experience recurrent, sometimes life-threatening, decompensation episodes that can often require hospitalisation [6]. Triheptanoin is a highly purified, synthetic medium odd-chain (C7) triglyceride that can bypass long-chain oxidation [7]. It is approved in the United States (US) as a source of calories and fatty acids in patients with LC-FAOD [8], but is only available as a part of compassionate use programs in other countries [9, 10].

A randomised clinical study demonstrated improved cardiac structure and function (at rest and during exercise) with triheptanoin versus an even-chain triglyceride (trioctanoin) for 4 months [11]; other studies showed a reduction in the annual rate and duration of major clinical events (i.e. rhabdomyolysis, hypoglycaemia and cardiomyopathy) compared with the pre-treatment period [7, 9]. Similarly, retrospective observational studies have reported a decreased incidence of major clinical events and lower hospitalisation rates with triheptanoin compared with MCT oil [12, 13], as well as stabilisation of cardiac function in patients with paediatric cardiomyopathy [14].

In this study, we retrospectively examined the effectiveness and safety of triheptanoin treatment compared with MCT oil in a cohort of patients with LC-FAOD in Italy.

## Methods

### Study design and patients

In this retrospective nationwide study, data were collected from patients who were diagnosed with LC-FAOD (the molecular variants detected in the different genes were: HDBHA: Glu510Gln; HDBHB: Thr69Ile, Gly427Glu, and Gly301Ser; CPT2: Tyr628Ser, His555Gln, and Ser113Leu; ACADVL: Gly185Ser, Val283Ala, His335Pro). Patients were treated with oral triheptanoin (provided at a mean dose of 1.5 ± 0.9 g/kg/day in four divided administrations) as part of an Agenzia Italiana del Farmaco (AIFA)-funded programme at five centres in Italy between 2018 and 2022. Patients were observed for a maximum of 4 years of triheptanoin treatment.

Ethics approval for this study was obtained from the ethics committee of AOU Citta della Salute e della Scienza di Torino, Italy (0069665–109/2023).

### Outcome measures

The following data were retrospectively collected from medical records: LC-FAOD diagnosis, age at diagnosis, method of diagnosis, age at the time of study enrolment, weight, height, body mass index (BMI) and details of treatment with MCT oil and subsequent triheptanoin treatment (i.e. duration, daily dose and percentage of total daily calories). Clinical outcome measures were the number of intercurrent catabolic episodes, the number of metabolic decompensation episodes requiring hospitalisation or intensive care unit (ICU) admissions, the number of hospitalisations per year (annualised hospitalisation rate) and days per hospitalisation, both during standard MCT therapy and during subsequent triheptanoin treatment. Creatine kinase (CK) levels (during and outside of metabolic decompensation episodes) and adverse effects during treatment were also assessed.

### Statistical analysis

Descriptive statistics were used for all analyses, and data are reported as mean ± standard deviation (SD). The Shapiro–Wilk test was used to check normal distribution of data. Differences between groups were established with the Student’s t-test or Wilcoxon–Mann–Whitney U test for parametric and non-parametric variables, respectively. The level of statistical significance for all calculations was taken as a two-tailed *p*-value less than 0.05.

Statistical analyses were conducted using IBM SPSS Statistics for Windows, version 28.0 (IBM Corp., Armonk, NY, USA).

## Results

### Patient characteristics

Nine patients (four males and five females) with LC-FAOD were treated with triheptanoin between 2018 and 2022 and were included in this analysis. Four patients had a diagnosis of MTPD, three had CPT2D and two had VLCADD (Table 1). The diagnosis was established by clinical symptoms alone in six patients, clinical symptoms plus newborn screening (NBS) by tandem mass spectrometry in two patients and NBS alone in one patient. In all patients, diagnosis of LC-FAOD was genetically confirmed. The mean age at diagnosis was 1.5 ± 1.8 years. At the time of study enrolment, patients had a mean age of 11.1 ± 4.5 years and a mean BMI z-score of 0.09 ± 1.00.

**Table 1:** Characteristics of nine patients with long-chain fatty acid oxidation disorders

Patient	LC-FAOD diagnosis	Age at diagnosis (years)	Diagnosis method	Current age (years)	Current weight (kg)	Current height (cm)	Current BMI z-score
1	MTPD	0.1	NBS + clinical symptoms	7.4	24	118	1
2	MTPD	1.0	Clinical symptoms	5.0	17	112	–1.2
3	CPT2D	0.6	Clinical symptoms	18.2	66	185	–1.1
4	MTPD	0.5	Clinical symptoms	12.0	41	155	–0.6
5	CPT2D	4.0	Clinical symptoms	16.0	71	178	0.6
6	CPT2D	5.0	Clinical symptoms	8.0	25	124	0.3
7	VLCADD	2.0	Clinical symptoms	15.0	63	164	1.2
8	MTPD	0.01	NBS + clinical symptoms	9.0	32	146	–0.7
9	VLCADD	0.3	NBS	8.9	31	128	1.3

*BMI* body mass index, *CPT2D* carnitine palmitoyl transferase 2 deficiency, *LC-FAOD* long-chain fatty acid oxidation disorders, *MTPD* mitochondrial trifunctional protein deficiency, *NBS* newborn screening, *VLCADD* very long-chain acyl-CoA dehydrogenase deficiency

### Treatment regimens

Prior to starting triheptanoin treatment, the patients received MCT oil for a mean of 7.0 ± 4.5 years. The mean dose of MCT oil was 1.7 ± 0.8 g/kg/day in four divided administrations, accounting for 21 ± 9% of the total daily caloric intake (Table 2). Triheptanoin treatment was started at between 2.7 and 16 years of age and was continued for a mean of 2.2 ± 0.9 years. Triheptanoin was provided at a mean dose of 1.5 ± 0.9 g/kg/day in four divided administrations, accounting for 24 ± 9% of the total daily caloric intake.

**Table 2:** Comparison of MCT oil versus triheptanoin in nine patients with long-chain fatty acid oxidation disorders

Patient	MCT oil treatment					Triheptanoin				
	**Treatment duration (years)**	**Age during treatment (years)**	**Hypolipidic diet, mean fat (g/kg/day)**	**Mean MCT dose (g/kg/day)**	**Calories from MCT (%)**	**Treatment duration (years)**	**Age during treatment (years)**	**Hypolipidic diet, mean fat (g/kg/day)**	**Mean triheptanoin dose (g/kg/day)**	**Calories from triheptanoin (%)**
1	3.2	0.1–3.3	2.6	2.6	22	4.0	3.3–7.3	2.8	2.4	30
2	1.7	1.0–2.7	2.5	2.5	30	2.3	2.7–5.0	3.2	2.9	32
3	15.4	0.6–16.0	0.9	0.5	10	2.5	16.0–18.5	1.0	0.7	23
4	10.0	0.5–11.0	–	1.0	13	1.0	11.0–12.0	–	0.5	10
5	6.0	7.0–13.0	–	0.5	8	3.0	13.0–16.0	1.6	1.1	29
6	1.0	5.0–6.0	–	2.0	24	2.0	6.0–8.0	2.1	0.8	10
7	11.0	2.0–13.0	–	2.0	26	2.0	13.0–15.0	1.7	1.0	34
8	7.0	0–7.0	–	1.7	20	2.0	7.0–9.0	–	2.5	26
9	7.0	1.0–8.0	2.3	2.3	36	1.0	8.0–9.0	2.6	1.3	21

*MCT* Medium-chain triglycerides

### Clinical events

**Fig. 1:**
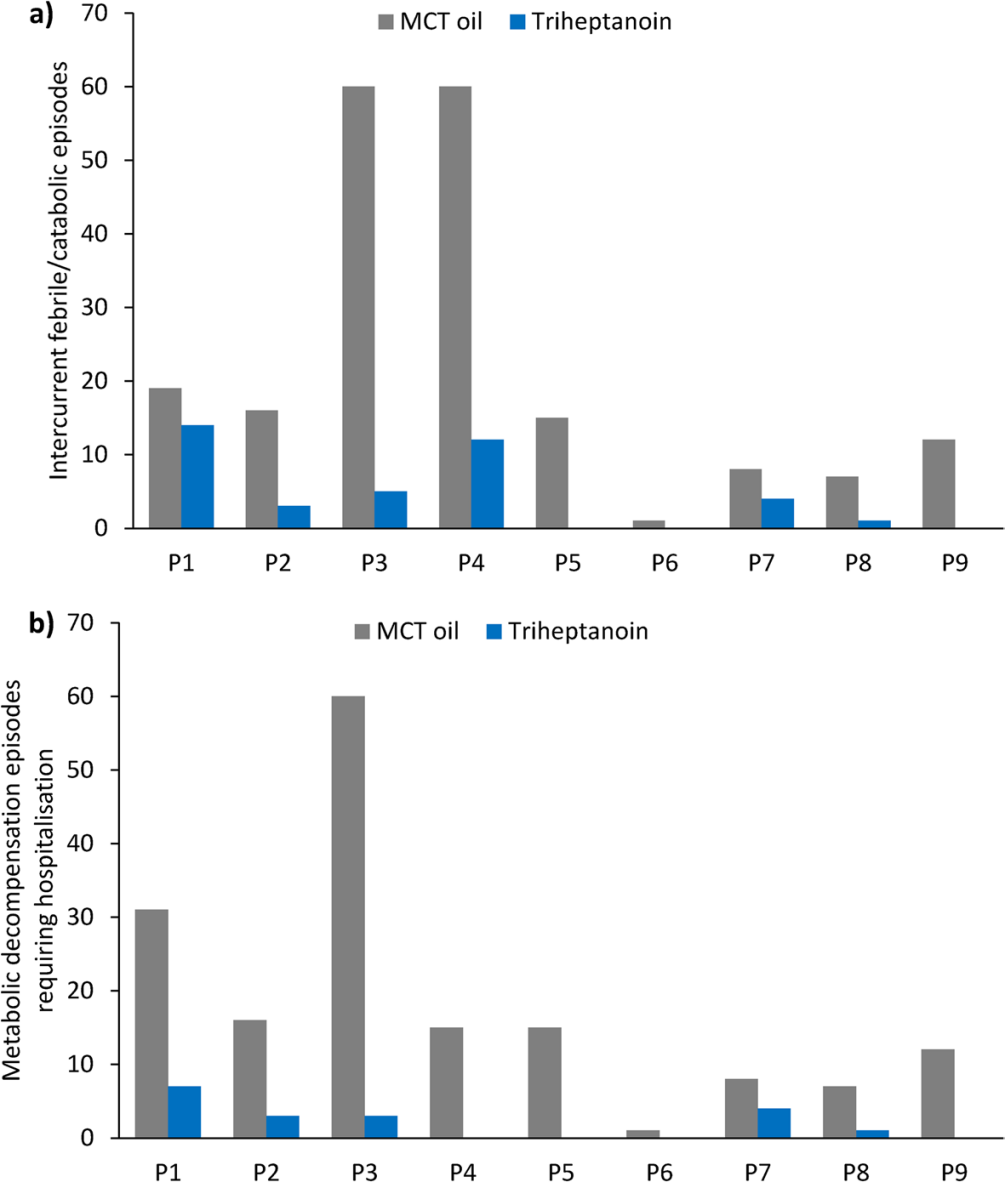
The number of **a** intercurrent febrile/catabolic episodes and **b** metabolic decompensation episodes requiring hospitalisation during medium-chain triglyceride (MCT) oil and subsequent triheptanoin treatment in each patient (P)

As shown in Fig. 1a, each patient had a significant reduction in the total number of intercurrent catabolic episodes during triheptanoin therapy compared with prior MCT oil (triheptanoin 4.3 ± 5.3 vs MCT 22.0 ± 22.2; *p* = 0.034). Similarly, all nine patients had a significant reduction in the number of metabolic decompensation episodes requiring hospitalisation after switching from MCT oil to triheptanoin treatment (triheptanoin 2.0 ± 2.5 vs MCT 18.3 ± 17.7; *p* = 0.014; Fig. 1b). During treatment with MCT oil, three patients required four ICU admissions for metabolic decompensation (i.e. rhabdomyolysis without hypoglycaemia or cardiomyopathy), while no ICU admissions were required during triheptanoin treatment.

**Fig. 2:**
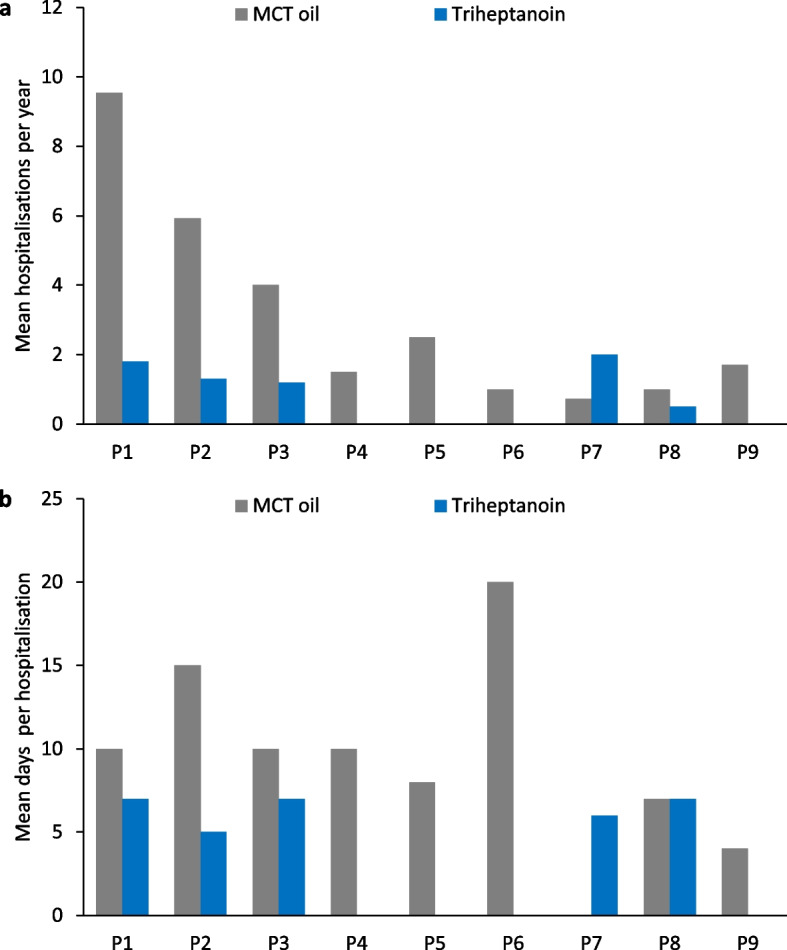
Mean number of **a** hospitalisations per year and **b** days per hospitalisation during medium-chain triglyceride (MCT) oil and subsequent triheptanoin treatment in each patient (P)

**Fig. 3:**
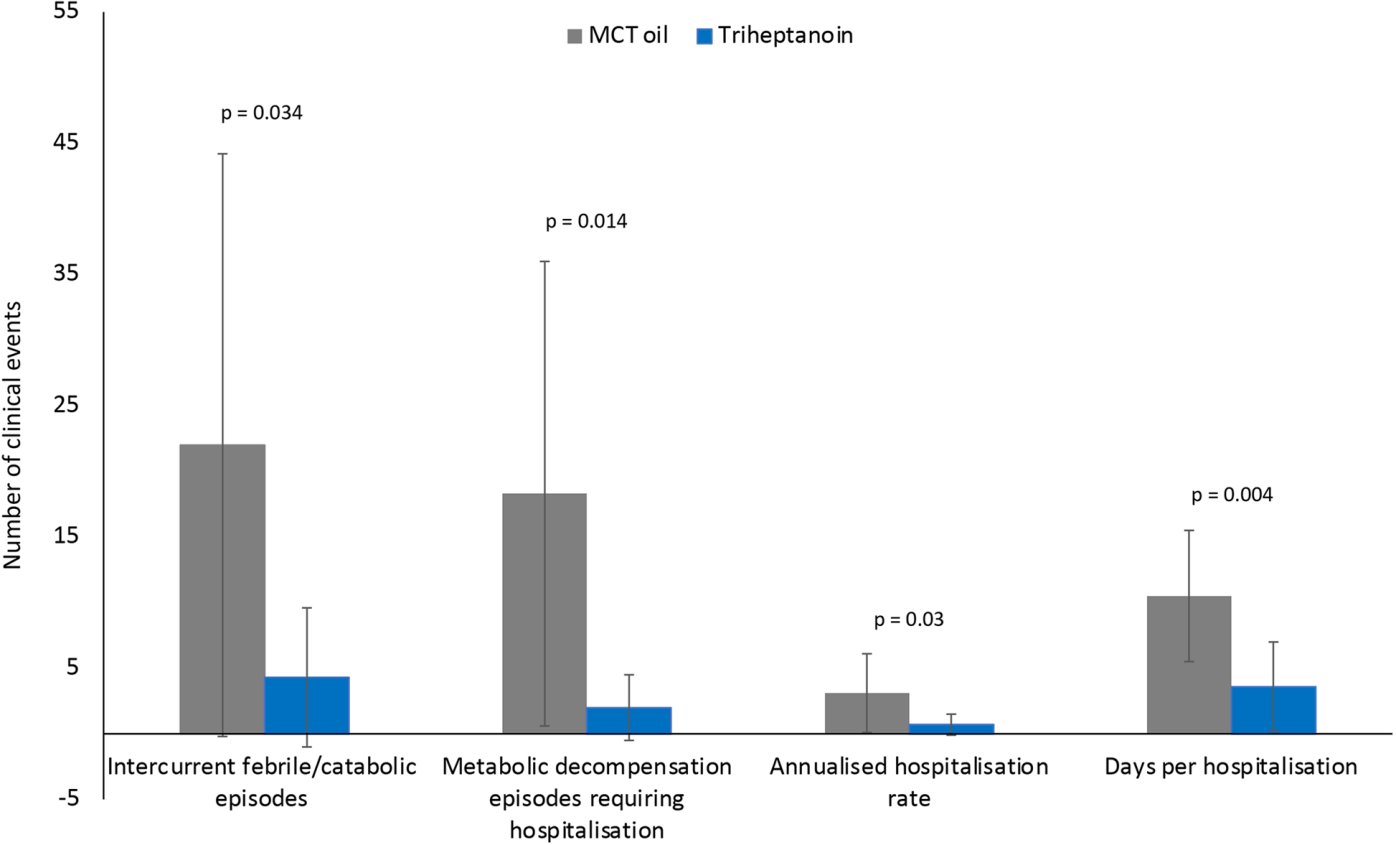
Comparison of the mean number of clinical events and hospitalisations during medium-chain triglyceride (MCT) oil and subsequent triheptanoin treatment in each patient (P)

Eight of nine patients had a reduction in the annualised hospitalisation rate after switching from MCT oil to triheptanoin treatment (triheptanoin 0.7 ± 0.8 vs MCT 3.1 ± 3.0 per year; *p* = 0.03; Fig. 2a), and seven had a reduction in the number of days per hospitalisation (triheptanoin 3.6 ± 3.4 vs MCT 10.5 ± 5.0; *p* = 0.004; Fig. 2b). There were significant improvements in the number and duration of clinical events after triheptanoin therapy (Fig. 3).

### Creatine kinase levels

**Fig. 4:**
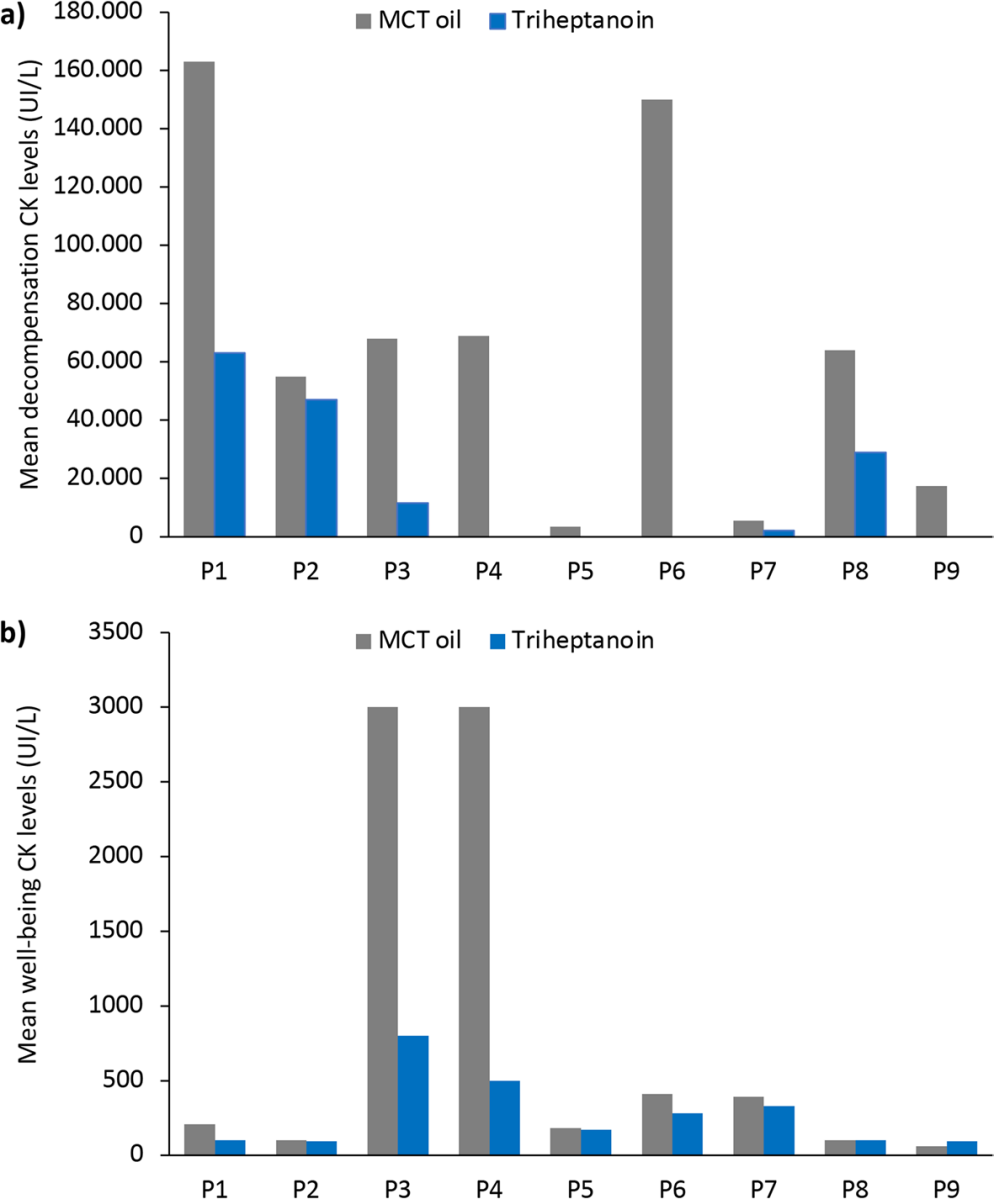
Mean **a** decompensation and **b** well-being creatine kinase (CK) levels during medium-chain triglyceride (MCT) oil and subsequent triheptanoin treatment in each patient (P)

Each patient showed a reduction in mean CK levels during metabolic decompensation after switching from MCT oil to triheptanoin treatment (Fig. 4a). Overall, mean CK levels during decompensation decreased from 66,178 ± 57,565 IU/L to 30,550 ± 24,958 IU/L during triheptanoin treatment, although this difference was not statistically significant (*p* = 0.218). One patient experienced two severe episodes of rhabdomyolysis (with concomitant high transaminases) during treatment with MCT oil that required continuous veno-venous haemodialysis. There were no occurrences of hypoketotic hypoglycaemia, cardiomyopathy, muscle weakness, hyperammonaemia (or Reye-like syndrome) or hepatomegaly during triheptanoin treatment.

Mean well-being CK levels (i.e. outside metabolic decompensation episodes) were lower with triheptanoin treatment versus MCT oil for seven patients (Fig. 4b), with a mean CK level for all patients of 828 ± 1238 UI/L during MCT oil treatment vs 274 ± 242 UI/L during triheptanoin treatment (*p* = 0.207).

### Safety

Two patients reported adverse effects during MCT oil treatment: diarrhoea in Patient 3 and epigastric pain in Patient 7, which resolved after the administration of a proton pump inhibitor. One patient (Patient 3) had adverse effects during triheptanoin treatment (epigastric pain and diarrhoea).

## Discussion

In this study, nine patients with LC-FAOD received triheptanoin treatment (accounting for 10–34% of the total daily caloric intake) for a mean of 2.2 years after switching from standard therapy with MCT oil (accounting for 8–36% of the total daily caloric intake) for a mean of 7.0 years. The focus of long-term nutritional management in LC-FAOD is to use an energy source other than long-chain fatty acids to provide sufficient daily calories [15]. Dietary management usually involves avoiding prolonged fasting, restriction of LCTs, a modest increase in carbohydrates and MCT supplementation as an alternative form of energy to LCTs [5, 6, 15].

MCTs consist of even-chain fatty acids, are 6–12 carbons long, do not require L-carnitine for mitochondrial transport and do not require oxidation by long-chain acyl-coenzyme A (CoA) hydrogenase enzymes [15], thereby bypassing the metabolic defects in LC-FAOD [6]. The recommended limit for long-chain fatty acid intake ranges from 10% of total daily calories in patients with severe forms of LC-FAOD to 45% in infants with mild disease [15].

Triheptanoin represents an alternative to MCT that has been associated with improved clinical outcomes in patients with LC-FAOD [7, 9, 11, 16, 17]. Triheptanoin consists of odd-chain fatty acids (C7) with anaplerotic properties (different from even-chain variants), which are metabolised through β-oxidation to acetyl-CoA [15]. Since patients with LC-FAOD do not properly oxidise fat to acetyl-CoA, there is an impairment of the tricarboxylic cycle and consequent lack of ketone body formation. Triheptanoin is a source of acetyl-CoA and propionyl-CoA, resulting in increased substrate availability in the tricarboxylic acid cycle [15]. The US prescribing information recommends providing up to 35% of total daily calories from triheptanoin, divided into at least four administrations per day [8]. A retrospective observational study conducted in the US reported that most patients survived the initial trigger event (e.g. severe infection) and demonstrated both short- and long-term improvements in manifestations of LC-FAOD following the initiation of triheptanoin therapy [17].

The current study found a significant reduction in the number of intercurrent catabolic episodes once patients switched from MCT oil to triheptanoin treatment. Enzyme deficiencies in the metabolic pathway that converts LCTs into energy can lead to depleted energy production, resulting in severe acute metabolic crises, such as rhabdomyolysis, hypoglycaemia and cardiomyopathy [18], which may lead to hospitalisation, ICU admission or premature death. In this study, metabolic decompensation episodes requiring hospitalisation were markedly reduced once triheptanoin treatment was started. Similarly, a previous study of three US patients with LC-FAOD reported fewer hospitalisations related to metabolic crises, as well as lower annualised rates and durations of hospitalisation, after the start of triheptanoin treatment [19]. In the current study, we observed a 77.4% reduction in the annualised hospitalisation rate during the triheptanoin treatment period, in line with the final results of a previous open-label extension study, which found an 85% reduction in this outcome measure [16]. In addition, patients with LC-FAOD who received triheptanoin in the previous study had reduced rates of hospitalisation due to rhabdomyolysis, cardiomyopathy and hypoglycaemia; these beneficial effects were sustained with long-term triheptanoin treatment [16].

CK levels, both during metabolic decompensation and steady-state conditions, were lower with triheptanoin versus MCT oil, although these differences did not reach statistical significance. These findings are consistent with those of a similar retrospective observational study of 12 patients with LC-FAOD in Austria, in which the total number of hospitalisation days decreased by 82% and the episodes of elevated CK levels (i.e. > 500 UI/L) decreased by 45% after initiation of triheptanoin (mean treatment duration 5.3 years) compared with the pre-treatment period [13]. Similar to our study, rhabdomyolysis also occurred in one patient during MCT oil therapy, but there were no cases of rhabdomyolysis recorded with triheptanoin treatment in the Austrian study [13]. Another retrospective US study of 52 patients with LC-FAOD reported that episodes of rhabdomyolysis requiring hospital admission were reduced from 85 to 31% after switching from MCT to triheptanoin treatment [20].

Current guideline recommendations specify that patients with severe LC-FAOD should receive their total caloric intake from MCT [15]. However, high-dose administrations of MCT may cause gastrointestinal symptoms (e.g. cramping, diarrhoea and vomiting) [21]. In our study, the only treatment-related adverse effects with triheptanoin were gastrointestinal in nature (i.e. epigastric pain and diarrhoea) and were similar to those observed during MCT treatment. Although triheptanoin is generally well tolerated [17, 22], the documented adverse effects in other studies were also predominantly mild gastrointestinal symptoms (e.g. diarrhoea, abdominal or gastrointestinal pain, nausea and vomiting, abdominal distension and flatulence) [10, 16].

In this study, molecular confirmation was obtained for all patients either diagnosed by NBS or clinically. To this purpose, the use of next generation sequencing can be useful to provide counseling to the family on genetic diseases, establishing the risk of recurrence and advancing potential genotype–phenotype correlations [23, 24].

The limitations of this study include its retrospective, open-label and uncontrolled design, and the relatively small and heterogeneous study population. Since the total daily caloric intake provided by MCT and triheptanoin treatment differed between patients, a dose-dependent effect on clinical outcomes could not be ruled out.

## Conclusions

Treatment with triheptanoin provided significant improvement of clinical outcomes compared with standard MCT therapy in patients with LC-FAOD, with decreases in intercurrent catabolic episodes, metabolic decompensation episodes requiring hospitalisation and the annualised rate and duration of hospitalisations in these patients. Triheptanoin treatment was well tolerated, with few adverse effects. These results highlight that triheptanoin is a safe and more effective treatment option compared with MCT in patients with LC-FAOD.

## Data Availability

The datasets used and/or analysed during the current study are available from the corresponding author on reasonable request.

## Abbreviations

AIFA: Agenzia Italiana del Farmaco
BMI: Body-mass index
CACTD: Carnitine-acylcarnitine translocase deficiency
CK: Creatine kinase
CoA: Coenzyme A
CPT1D: Carnitine palmitoyltransferase type 1 deficiency
CPT2D: Carnitine palmitoyltransferase type 2 deficiency
ICU: Intensive care unit
LC-FAOD: Long-chain fatty acid oxidation disorders
LCHADD: Long-chain hydroxyacyl-coenzyme-A dehydrogenase deficiency
LCT: Long-chain triglyceride
MCT: Medium-chain triglyceride
MTPD: Mitochondrial trifunctional protein deficiency
SD: Standard deviation
US: United States
VLCADD: Very long-chain acyl-coenzyme A dehydrogenase deficiency

## References

[1] Knottnerus SJG, Bleeker JC, Wust RCI, Ferdinandusse S, Ijlst L, Wijburg FA, et al. Disorders of mitochondrial long-chain fatty acid oxidation and the carnitine shuttle. Rev Endocr Metab Disord. 2018;19:93–106. 29926323 10.1007/s11154-018-9448-1 PMC6208583

[2] Merritt JL 2nd, Norris M, Kanungo S. Fatty acid oxidation disorders. Ann Transl Med. 2018;6:473. 30740404 10.21037/atm.2018.10.57 PMC6331364

[3] Sklirou E, Alodaib AN, Dobrowolski SF, Mohsen AA, Vockley J. Physiological perspectives on the use of triheptanoin as anaplerotic therapy for long chain fatty acid oxidation disorders. Front Genet. 2020;11: 598760. 33584796 10.3389/fgene.2020.598760 PMC7875087

[4] Vockley J. Long-chain fatty acid oxidation disorders and current management strategies. Am J Manag Care. 2020;26:S147–54. 32840329 10.37765/ajmc.2020.88480 PMC9850137

[5] Spiekerkoetter U, Lindner M, Santer R, Grotzke M, Baumgartner MR, Boehles H, et al. Treatment recommendations in long-chain fatty acid oxidation defects: consensus from a workshop. J Inherit Metab Dis. 2009;32:498–505. 19452263 10.1007/s10545-009-1126-8

[6] Baker JJ, Burton BK. Diagnosis and clinical management of long-chain fatty-acid oxidation disorders: a review. touchREV Endocrinol. 2021;17:108–11. 35118456 10.17925/EE.2021.17.2.108 PMC8676101

[7] Vockley J, Burton B, Berry G, Longo N, Phillips J, Sanchez-Valle A, et al. Effects of triheptanoin (UX007) in patients with long-chain fatty acid oxidation disorders: results from an open-label, long-term extension study. J Inherit Metab Dis. 2021;44:253–63. 32885845 10.1002/jimd.12313 PMC7891391

[8] US Food and Drug Administration. Dojolvi^TM^ (triheptanoin) oral liquid: prescribing information. https://www.accessdata.fda.gov/drugsatfda_docs/label/2020/213687s000lbl.pdf (2020). Accessed 17 Apr 2023.

[9] Vockley J, Burton B, Berry GT, Longo N, Phillips J, Sanchez-Valle A, et al. Results from a 78-week, single-arm, open-label phase 2 study to evaluate UX007 in pediatric and adult patients with severe long-chain fatty acid oxidation disorders (LC-FAOD). J Inherit Metab Dis. 2019;42:169–77. 30740733 10.1002/jimd.12038 PMC6348052

[10] Vockley J, Burton B, Berry GT, Longo N, Phillips J, Sanchez-Valle A, et al. UX007 for the treatment of long chain-fatty acid oxidation disorders: safety and efficacy in children and adults following 24 weeks of treatment. Mol Genet Metab. 2017;120:370–7. 28189603 10.1016/j.ymgme.2017.02.005

[11] Gillingham MB, Heitner SB, Martin J, Rose S, Goldstein A, El-Gharbawy AH, et al. Triheptanoin versus trioctanoin for long-chain fatty acid oxidation disorders: a double blinded, randomized controlled trial. J Inherit Metab Dis. 2017;40:831–43. 28871440 10.1007/s10545-017-0085-8 PMC6545116

[12] Vockley J, Marsden D, McCracken E, DeWard S, Barone A, Hsu K, et al. Long-term major clinical outcomes in patients with long chain fatty acid oxidation disorders before and after transition to triheptanoin treatment—a retrospective chart review. Mol Genet Metab. 2015;116:53–60. 26116311 10.1016/j.ymgme.2015.06.006 PMC4561603

[13] Zöggeler T, Stock K, Jörg-Streller M, Spenger J, Konstantopoulou V, Hufgard-Leitner M, et al. Long-term experience with triheptanoin in 12 Austrian patients with long-chain fatty acid oxidation disorders. Orphanet J Rare Dis. 2021;16:28. 33446227 10.1186/s13023-020-01635-x PMC7807521

[14] Vockley J, Charrow J, Ganesh J, Eswara M, Diaz GA, McCracken E, et al. Triheptanoin treatment in patients with pediatric cardiomyopathy associated with long chain-fatty acid oxidation disorders. Mol Genet Metab. 2016;119:223–31. 27590926 10.1016/j.ymgme.2016.08.008 PMC5083220

[15] Rohr F. Nutrition management of fatty acid oxidation disorders. In: Bernstein LE, Rohr F, van Calcar S, editors. Nutrition management of inherited metabolic diseases. Cham: Springer; 2022. p. 325–35.

[16] Vockley J, Burton BK, Berry G, Longo N, Phillips J, Sanchez-Valle A, et al. Triheptanoin for the treatment of long-chain fatty acid oxidation disorders: final results of an open-label, long-term extension study. J Inherit Metab Dis. 2023;46:943–55. 37276053 10.1002/jimd.12640

[17] Vockley J, Enns GM, Ramirez AN, Bedrosian CL, Reineking B, Lu X, et al. Response to triheptanoin therapy in critically ill patients with LC-FAOD: report of patients treated through an expanded access program. Mol Genet Metab. 2022;136:152–62. 35459555 10.1016/j.ymgme.2022.04.001

[18] Vockley J, Longo N, Madden M, Dwyer L, Mu Y, Chen C-Y, et al. Dietary management and major clinical events in patients with long-chain fatty acid oxidation disorders enrolled in a phase 2 triheptanoin study. Clin Nutr ESPEN. 2021;41:293–8. 33487279 10.1016/j.clnesp.2020.11.018 PMC8567087

[19] Norris MK, Scott AI, Sullivan S, Chang IJ, Lam C, Sun A, et al. Tutorial: Triheptanoin and nutrition management for treatment of long-chain fatty acid oxidation disorders. J Parenter Enteral Nutr. 2021;45:230–8. 10.1002/jpen.2034 33085788

[20] Roe CR, Brunengraber H. Anaplerotic treatment of long-chain fat oxidation disorders with triheptanoin: review of 15 years experience. Mol Genet Metab. 2015;116:260–8. 26547562 10.1016/j.ymgme.2015.10.005 PMC4712637

[21] Liu YM. Medium-chain triglyceride (MCT) ketogenic therapy. Epilepsia. 2008;49:33–6. 19049583 10.1111/j.1528-1167.2008.01830.x

[22] Shirley M. Triheptanoin: first approval. Drugs. 2020;80:1595–600. 32897506 10.1007/s40265-020-01399-5 PMC7575481

[23] Schierz IAM, Serra G, Antona V, Persico I, Corsello G, Piro E. Infant developmental profile of Crisponi syndrome due to compound heterozygosity for CRLF1 deletion. Clin Dysmorphol. 2020;29:141–3. 32433043 10.1097/MCD.0000000000000325

[24] Serra G, Antona V, Insinga V, Morgante G, Vassallo A, Placa S, et al. Carnitine palmitoyltransferase II (CPT II) deficiency responsible for refractory cardiac arrhythmias, acute multiorgan failure and early fatal outcome. Ital J Pediatr. 2024;50:67. 38616285 10.1186/s13052-024-01632-x PMC11017661

